# The Trial of New Forms of Treatment

**Published:** 1901-01-19

**Authors:** 


### THE TRIAL OF NEW FORMS OF TREATMENT.

THE story told in a recent number of the New York *Medical News* concerning the trial which had been given in St. Luke's Hospital in that city to a much vaunted method for the treatment of consumption by electricity, should teach those who are responsible for the management of great public institutions to be careful and to take much heed before stepping aside in the slightest degree from their proper work, or using hospitals for any but their proper purposes. The opportunities which exist in large hospitals for putting various remedies or supposed remedies to the test are so ample that there is always some one ready to suggest that it is a reasonable and a proper thing to make a trial of some of the hundred and one quack remedies, the efficacy of which is vouched for with such an engaging semblance of truth by their inventors or proprietors; and, indeed, the great impulsive and unreasoning public is ready enough to declare that but for the prejudice and narrowmindedness of our profession all sorts of fine "cures" would be at the disposal of hospital patients which at present are kept outside the gates. Hence we need not be surprised that now and again we hear of trials of this sort being made, and lest they should be made too often we draw attention to what is stated to have happened lately in New York. With singular generosity, we are told, St. Luke's Hospital consented to experiment upon a method of treatment by electricity which claimed to have had extraordinary success in dealing with consumption. The treatment was given to certain patients regularly, accurately, and systematically, control experiments being carried on at the same time, and complete bacteriological examinations being undertaken to see whether or no any improvement was produced, and after three months of careful observations the physicians of the hospital gave it as their unbiassed opinion that there was nothing in it. One might perhaps have imagined that this would have brought the matter to an end. But not at all. So far from this giving its quietus to the treatment in question, we are told that the fact of its having been used at St. Luke's is being quoted by its advocates as conclusive evidence that the method is vouched for and practised by the most reputable hospital in the country. It is added that references to these experiments have been made even at medical meetings and in medical literature, always with the assumption that the test experiments were successful, notwithstanding that these assumptions have been denied and the promulgators of them have been snubbed. Such snubbings, however, "make no difference," we are told, "to those who are sounding the clarion for the cure of tuberculosis by electricity. Their point has been gained," for the sanction of use in a well-known hospital has been given to this advertised consumption cure, and no one hears the denials of its utility made by the physicians who watched the experiment. We may properly take the warning to heart. Pressure is now and again brought to bear upon hospital managers to get this or that thing tried in hospitals, and sometimes the proposed modes of treatment appear to be surrounded with such a halo of probability and to carry with them such a semblance of scientific truth, that it is difficult to refuse. But we must harden our hearts, and search strictly into the proofs offered by the inventors of strange remedies before allowing them to be tried in hospitals.

